# Mother-preterm infant interactions at 3 months of corrected age: influence of maternal depression, anxiety and neonatal birth weight

**DOI:** 10.3389/fpsyg.2015.01234

**Published:** 2015-09-01

**Authors:** Erica Neri, Francesca Agostini, Paola Salvatori, Augusto Biasini, Fiorella Monti

**Affiliations:** ^1^Department of Psychology, University of BolognaBologna, Italy; ^2^Paediatric and Neonatal Intensive Care Unit, Bufalini Hospital, CesenaItaly

**Keywords:** maternal depression, maternal anxiety, birth weight, mother–infant interaction, preterm birth

## Abstract

Maternal depression and anxiety represent risk factors for the quality of early mother-preterm infant interactions, especially in the case of preterm birth. Despite the presence of many studies on this topic, the comorbidity of depressive and anxious symptoms has not been sufficiently investigated, as well as their relationship with the severity of prematurity and the quality of early interactions. The Aim of this study was to evaluate the quality of early mother-infant interactions and the prevalence of maternal depression and anxiety comparing dyads of extremely low birth weight (ELBW) and very low birth weight (VLBW) preterm infants with full-term ones. Seventy seven preterm infants (32 ELBW; 45 VLBW) and 120 full term (FT) infants and their mothers were recruited. At 3 months of corrected age, 5 min of mother-infant interactions were recorded and later coded through the Global Ratings Scales. Mothers completed the Edinburgh Postnatal Depression Scale and Penn State Worry Questionnaire. Infant levels of development were assessed through the Griffiths Mental Development Scales. A relation emerged among the severity of prematurity, depression, anxiety, and the quality of interactions. When compared with the FT group, the ELBW interactions were characterized by high maternal intrusiveness and low remoteness, while the VLBW dyads showed high levels of maternal sensitivity and infant communication. Depression was related to maternal remoteness and negative affective state, anxiety to low sensitivity, while infant interactive behaviors were impaired only in case of comorbidity. ELBW’s mothers showed the highest prevalence of depressive and anxious symptoms; moreover, only in FT dyads, low maternal sensitivity, negative affective state and minor infant communication were associated to the presence of anxious symptoms. The results confirmed the impact of prematurity on mother–infant interactions and on maternal affective state. Early diagnosis can help to plan supportive interventions.

## Introduction

### Maternal Depression and Anxiety in the Postnatal Period

Postnatal depression has been widely recognized as a significant risk factor for woman’s health, baby’s development and the quality of mother–infant interactions (O’Hara and Swain, 1996; Martins and Gaffan, 2000; Guedeney and Jeammet, 2001; Righetti-Veltema et al., 2002; Beebe et al., 2012; Piteo et al., 2012). Recent studies have underlined that postnatal depression is associated to infants’ level of oxytocin, imparing their response to stress (Apter-Levy et al., 2013; Feldman, 2015; Pratt et al., 2015). Over the last decade, literature has highlighted that anxiety is also common and often comorbid with depression during the perinatal period (Josefsson et al., 2001; Austin et al., 2010; Field et al., 2010; Beebe et al., 2011; Figueiredo and Conde, 2011).

The prevalence of maternal depression and anxiety in the postpartum period is about 15–20% (O’Hara and Swain, 1996; Guedeney and Jeammet, 2001; Reck et al., 2008; Seymour et al., 2015) and can increase in women that experienced miscarriage, still birth or preterm birth (Davis et al., 2003; Voegtline et al., 2010; Padovani et al., 2011).

When undiagnosed, postnatal depression and anxiety might have long-term effects both on the mother and on the infant (Kersting et al., 2004; Murray et al., 2011) and literature shows how both depression and anxiety impact on the quality of mother–infant interactions, which appear as less synchronous and coordinated than those of non-depressed or non-anxious mother-infant dyads (Mertesacker et al., 2004; Nicol-Harper et al., 2007; Zelkowitz et al., 2009). Depressed mothers appear to be less sensitive and responsive than non-depressed ones; indeed, they are more remote or intrusive during the interaction with their babies, who, in return, tend to react to maternal behavior with passivity or distress (Field, 1984, 1995; Diego et al., 2006; Feldman and Eidelman, 2007). Anxiety has a similar effect on the quality of mother–child interactions, even though the interactive behavior of these dyads has been less explored so far. In particular, recent literature focused on the cognitive components of anxiety and on the specific worries linked to the perinatal period (Murray et al., 2007; Stein et al., 2012).

### Maternal Depression and Anxiety after Preterm Birth

Prematurity is defined as the condition of all babies born alive before the 37th week of pregnancy has been completed (World Health Organization [WHO], 2012). It has been estimated that more than 1 out of 10 babies around the world are born preterm and prematurity is one of the leading causes of infant mortality, pediatric morbidity and long-term disability (Aarnoudse-Moens et al., 2009; McCormick et al., 2011). Along with the objective risk for the baby’s health, preterm birth is an unexpected and stressful event for the parents, which may leave them disoriented and frightened (Lasiuk et al., 2013). Parents might experience feelings of guilt, grief and recurrent concerns about their baby’s survival and health (Mendelsohn, 2005; Korja et al., 2009, 2010; Shah et al., 2011; Lasiuk et al., 2013). The persistent worries and the stress experienced can be so intense as to satisfy the criteria of post-traumatic stress disorder (DeMier et al., 2000; Pierrehumbert et al., 2003; Kersting et al., 2004).

The risk of developing anxiety and depression increases when the baby’s prematurity is more severe (Vigod et al., 2010). The prevalence of depression in mothers of very low birth weight (VLBW) infants ranges from 12 to 30% in the first 4 months post-partum (Padovani et al., 2004; Miles et al., 2007; Mehler et al., 2011; Gray et al., 2012). As for anxiety, literature showed that the rate of maternal anxiety ranges from 35 to 43% during the baby’s hospitalization in the NICU (Singer et al., 1999; Padovani et al., 2004, 2011) and from 12 to 26% after its discharge (Padovani et al., 2004; Rogers et al., 2013). However, to our knowledge, there is a lack of studies exploring the cognitive components of maternal anxiety in case of preterm birth. Similarly, the comorbidity between anxiety and depression has been poorly investigated in the maternal context.

### Mother–Infant Interactions and Preterm Birth

Recently, an increasing number of studies have focused on the impact of prematurity on early- interactions between mother and baby, finding that preterm dyads experience poorer and less synchronous interactions than full-term ones (Poehlmann and Fiese, 2001; Holditch-Davis et al., 2007; Korja et al., 2012). On the one hand, preterm babies appear as less active and responsive during the interaction with their mothers than full-term infants; this is due to their biological immaturity (Bozzette, 2007; Feldman and Eidelman, 2007). On the other hand, preterm infants’ mothers are generally reported as less sensitive and more intrusive than full term (FT) infants’ ones (Feldman, 2007a; Forcada-Guex et al., 2011).

However, literature on mother–child interactions in preterm dyads shows some inconsistencies (Bozzette, 2007; Korja et al., 2012). There is a lack of data exploring the relationship among the quality of early interactions, maternal depression, anxiety, and the severity of prematurity. To our knowledge, only Agostini et al. (2014) have partially investigated this relationship, finding that the quality of mother-preterm infant interactions could be impaired in specific ways both by the presence of maternal depression and by the severity of premature birth.

### Aim of the Study

The objective of the following study was to fulfill the gap existing in previous literature.

The general aim was to explore how the severity of prematurity, depression, and anxiety might impact on the quality of early interactions, therefore to conduct an explorative study. Specifically, we aimed at evaluating if the severity of birth weight was significantly associated to the quality of mother–infant interactions. Secondly, we investigated the influence of maternal depression/anxiety, considered both separately as well as their interaction, on interactive patterns (maternal and infant ones). With reference to anxiety, we specifically focused on detecting maternal worries, which are the cognitive components of anxiety and the key symptoms of generalized anxiety disorder (GAD; American Psychiatric Association [APA], 2000).

To this end, mother–infant interactions in preterm dyads were observed and compared to mother–infant interactions in FT dyads at 3 months post-partum. This time of the assessment was chosen, based on the evidence that the third month postpartum is a significant step for the co- construction of dyadic interactive patterns (Feldman, 2007b; Tronick, 2007) and for the detection of postnatal depression (Cox et al., 1987a; Cramer, 2000).

## Materials and Methods

### Participants

During the period March 2010-February 2013, all the mothers of preterm infants with birth weight under 1500 g and a gestational age <32 weeks, who had been hospitalized at the NICU of the Bufalini Hospital (Cesena, Italy), were asked to take part in the study. Only five mothers refused to participate. A total of 77 mother–infant dyads were recruited. The severity of their prematurity was evaluated according to their birth weight: 32 infants were extremely low birth weight (ELBW; under 1000 g) and 45 were VLBW (birth weight between 1000 and 1500 g).

During the period March 2011–August 2012, a psychologist met potential subjects for the control group in 36 antenatal classes held in Cesena. Each antenatal class was attended by 10–12 pregnant women, at the third trimester of pregnancy; ~30% in each group accepted to participate in the study voluntarily. All women were included in the sample as none of them had severe complications at delivery and gave birth to a healthy full-term baby. A total of 120 women were recruited (FT group).

Exclusion criteria for both groups were: infant chromosomal abnormalities, cerebral palsy, malformations and fetopathy, previous or present parents’ psychiatric illness and lack of fluency in Italian.

### Procedure

This study is part of a longitudinal research that followed mother- preterm infant dyads from 3 to 18 months of corrected age. The study protocol was approved by the Ethic Committee of the Department of Psychology (University of Bologna).

Mother–infant dyads were assessed at infant’s 3 months of age (corrected age for preterm infants). Mothers and their infants were met by a psychologist at the Laboratory of Psychodynamic of Development, Department of Psychology, University of Bologna, Italy.

All mothers completed a written consent form and a socio-demographic questionnaire. Perinatal data were collected for all dyads.

### Measures

A general quotient (GQ) of the infants’ development was assessed by means of the Griffiths Mental Development Scales-Revised version (GMDS-R for 0–2 years), a well-recognized measure for infant mental and psychomotor development (Griffiths, 1996). The GQ represents the mean score of 5 areas of development (Locomotor, Personal-Social, Hearing and Language, Eye and Hand Co-ordination, Performance). The GMDS-R has been largely used in Italian samples of preterm infants (Giannì et al., 2007; Monti et al., 2013; Agostini et al., 2014; Biasini et al., 2015b).

The presence of depressive symptoms in the postnatal period was investigated through the Edinburgh Postnatal Depression Scale (EPDS; Cox et al., 1987b), a self-report questionnaire. Mothers were asked to describe their mood during the previous 7 days by means of 10 items, each scoring between 0 to 3, with higher total scores indicating increasing distress. The Italian version (Benvenuti et al., 1999) showed good internal consistency (0.78). In the present study, EPDS was used both as a continuous and a categorical variable (depressed vs. non-depressed), with a cut-off value of 12/13 to screen for major depressive symptomatology, according to a previous Italian study (Agostini et al., 2015).

As to maternal anxiety, all mothers completed the Penn State Worry Questionnaire (PSWQ; Meyer et al., 1990), a self-report questionnaire designed to assess generalized pathological worries, considering the degree of excessiveness and of uncontrollability. It was developed to evaluate an individual’s disposition to worry, as well as the frequency of the condition, its excess or intensity, as well as the tendency for the person to worry generally and not in one or a small number of situations. PSWQ is composed of 16 items, rated on a Likert scale between 1 (Not at all typical of me) to 5 (Very typical of me). Eleven items are positively worded (e.g., “Once I start to worry, I can’t stop”) and five items are negatively worded (e.g., “I never worry about anything”). All negatively worded items are reverse scored and the sum of all the item scores gives a total that ranges from 16 to 80, where the higher the value, the higher the levels of pathological worry. PSWQ was previously used to detect the presence of anxious symptomatology in the perinatal period (Murray et al., 2007; Swanson et al., 2011; O’Connor et al., 2013). The Italian version of PSWQ suggests a cut-off score of 57 to discriminate anxious and non-anxious subjects and showed good internal consistency (0.85) (Morani et al., 1999). As for EPDS, in the present study we used both continuous and categorical scores (anxious vs. non-anxious mothers).

Infant and maternal interactive behaviors were coded by means of the Global Rating Scales (GRS) for Mother–Infant interaction (Murray et al., 1996a,b). Similarly to other rating systems (e.g., Cohn et al., 1986; Field et al., 1990), GRS are video-based assessments of the quality of mother-infant engagement in the 2–4 month postnatal period and have originally been developed to distinguish depressed and non-depressed mother–infant interactions for research purposes. Previous Authors often used GRS to discriminate a wide range of infant and maternal populations (e.g., clinical groups with schizophrenia, social adversity), showing good reliability (Riordan et al., 1999; Gunning et al., 2004; Grant et al., 2010; Costa and Figueiredo, 2011; Montirosso et al., 2012; Agostini and Murray, 2014) and validity in predicting the subsequent child’s performance (Murray et al., 1996a,b). As to the procedure, the mother was asked to sit opposite her baby, and to freely interact for 5 min without toys, as she usually would do at home. Video recordings of the episode were rated by a trained and expert rater (blind to maternal mood) on four maternal behavioral dimensions (Sensitivity, Intrusiveness, Remoteness, Signs of depression) and on three infant’s ones (Communicative, Inert, Distressed). All the dimensions are scored on a 5-point Likert scale, where 1 always corresponds to “poor” interactive maternal or infant behavior and 5 to most “optimal” behavior. A second rater coded ten videos randomly selected: the intra-class correlations showed acceptable reliability (mean = 0.75, range 0.68–0.88).

## Results

The infant and maternal socio-demographic characteristics are shown in **Table 1**.

**Table 1:** Infant and maternal characteristics.

	ELBW (*n* = 32)	VLBW (*n* = 45)	FT (*n* = 120)	*p* value
**Infant characteristics**		
Gestational age, weeks (mean ± *SD*, range)	27.52 ± 2.06, 24–31	29.70 ± 1.33, 26.86–32	39.93 ± 1.08, 37–41	<0.0005
Birth weight, grams, (mean ± *SD*, range)	823.00 ± 96.6, 599–985	1260.39 ± 162.7, 1005–1500	3476.57 ± 428.4, 2700–4780	<0.0005
Birth Length, cm (mean ± *SD*, range)	34.28 ± 2.47, 30.0–38.8	39.24 ± 2.99, 28.50–43.50	51.41 ± 2.08, 46.0–57.0	<0.0005
Length of hospitalization	60.93 ± 16.74, 36–92	37.23 ± 15.94, 10–89	2.09 ± 0.34, 2–4	<0.0005
Type of delivery,				<0.0005
Spontaneous (%)	25.0	22.5	78.4	
Cesarian section (%)	75.0	77.5	21.6	
Gender				0.153
Male (%)	51.6	66.7	50.0	
Female (%)	48.4	33.3	50.0	
GMDS general quotients (GQ), (mean ± *SD*, range)	104.09 ± 12.44	111.02 ± 8.48	111.98 ± 8.15	<0.0005
**Maternal characteristics**		
Maternal age, years (mean ± *SD*, range)	33.54 ± 5.46, 21–42	33.82 ± 5.91, 23–47	32.89 ± 5.02, 18–45	0.588
Education, years, (mean ± *SD*, range)	11.90 ± 3.75, 8–18	12.78 ± 3.19, 8–18	14.67 ± 3.20, 8–18	<0.0005
Parity				0.004
Nulliparous (%)	75.0	66.7	88.3	
Multiparous (%)	25.0	33.3	11.7	
Marital status				0.319
Married/cohabiting	96.9	97.8	92.3	
Other	3.1	2.2	7.7	

*ELBW, extremely low birth weight infants; VLBW, very low birth weight infants; FT, full term infants.*

The three groups showed significant differences regarding the following infant characteristics: gestational age, birth weight, birth length, length of hospitalization, and type of delivery (**Table 1**); since these variables are strictly linked to preterm birth, these results were expected. No differences emerged regarding the infants’ gender.

As to maternal variables, the three groups were homogeneous with relation to most of the variables, except for parity and level of education (**Table 1**): FT infants’ mothers, compared to ELBW and VLBW ones, were primiparous in a higher percentage [χ^2^(2) = 11.495; *p* = 0.003] and had a higher level of education [*F*(1,196) = 12.023; *p* < 0.0005; Bonferroni *post hoc*: *p* < 0.0005 and *p* = 0.003, respectively].

Moreover, the level of infant development, as measured by the GQ (GMDS-R) was significantly different among the three groups [*F*(2,196) = 9.69, *p* < 0.0005; **Table 1**]: Bonferroni’s *post hoc* analyses showed that ELBW infants had significantly lower scores than both VLBW and FT groups (*p* = 0.004 and *p* < 0.0005, respectively).

Specific analyses were run to control the effect of maternal parity, years of education and GQ on EPDS, PSWQ and GRS scores: parity did not show any significant influence, while education and level of development were significantly associated with dependent variables. Therefore, the years of education and GQ were always included as covariates in consecutive analyses in order to control their influence.

### Mother–Infant Interactions

The Univariate ANOVA was run for each GRS scale in order to analyze the main effects of birth weight, maternal depression and anxiety variables on interactive behaviors, also considering their possible interaction; GQ score and maternal education were always included as covariates (**Table 2**).

**Table 2:** Mother–infant interactive behaviors (GRS): differences among groups.

	Birth Weight			Maternal Depression		Maternal Anxiety		*F*					
	ELBW (*n* = 32)	VLBW (*n* = 45)	FT (*n* = 120)	Depressed (*n* = 20)	No-depressed (*n* = 177)	Anxious (*n* = 15)	No anxious (*n* = 182)	Birth weight	Maternal depression	Maternal anxiety y	Maternal depression × anxiety	Birth weight × maternal depression	Birth weight t × maternal anxiety y
**Maternal interactive dimensions**		
Sensitivity	3.37 ± 0.12	3.60 ± 0.13^b^	3.12 ± 0.14^b^	3.31 ± 0.12	3.38 ± 0.08	3.13 ± 0.13	3.51 ± 0.08	3.147*	0.633	4.355*	0.280	0.280	5.153*
Intrusiveness	4.50 ± 0.20^a^	4.25 ± 0.22	3.83 ± 0.22^a^	3.90 ± 0.17	3.76 ± 0.12	3.86 ± 0.19	3.82 ± 0.11	4.993*	0.382	0.028	0.244	0.244	0.138
Remoteness	3.42 ± 0.17^a^	3.86 ± 0.19	4.24 ± 0.19^a^	3.91 ± 0.20	4.52 ± 0.14	4.18 ± 0.22	4.20 ± 0.13	2.985*	7.389*	0.046	2.408	1.716	0.544
Signs of depression	3.98 ± 0.11^a^	4.02 ± 0.12^b^	3.56 ± 0.12^ab^	3.72 ± 0.11	3.98 ± 0.74	3.72 ± 0.12	3.94 ± 0.07	5.720**	5.373*	1.327	3.726*	0.625	3.445*
**Infant Interactive Dimensions**		
Communicative	2.85 ± 0.18	3.08 ± 0.20^b^	2.30 ± 0.20^b^	2.64 ± 0.17	2.81 ± 0.12	2.55 ± 0.20	2.85 ± 0.12	6.436*	3.641	0.205	6.041*	0.252	5.768*
Inert	3.11 ± 0.17	3.21 ± 0.18	2.93 ± 0.19	2.96 ± 0.16	3.21 ± 0.11	2.94 ± 0.18	3.19 ± 0.11	1.328	2.913	0.413	3.777*	0.324	1.347
Distressed	3.76 ± 0.13	3.82 ± 0.14	3.53 ± 0.15	3.67 ± 0.13	3.73 ± 0.09	3.68 ± 0.14	3.71 ± 0.09	1.577	0.504	0.009	1.073	0.027	1.636

*Scores are rated on 5-point scale (1 = poor, 5 = optimal). Values are mean ± *SD**

*^a^*p* < 0.05 for *post hoc* test (ELBW preterm dyads vs FT dyads).*

^b^*p* < 0.05 for *post hoc* test (VLBW preterm dyads vs FT dyads).

*p < 0.05.

**p < 0.005.

#### Birth Weight

The three groups showed significant differences on all the scales about maternal behaviors: Sensitivity [*F*(2,196) = 3.147; *p* = 0.045], Intrusiveness [*F*(2,196) = 4.993; *p* = 0.008], Remoteness [*F*(2,196) = 2.985; *p* = 0.050] and Signs of Depression [*F*(2,196) = 5.720; *p* = 0.004; **Table 2**].

**FIGURE 1:**
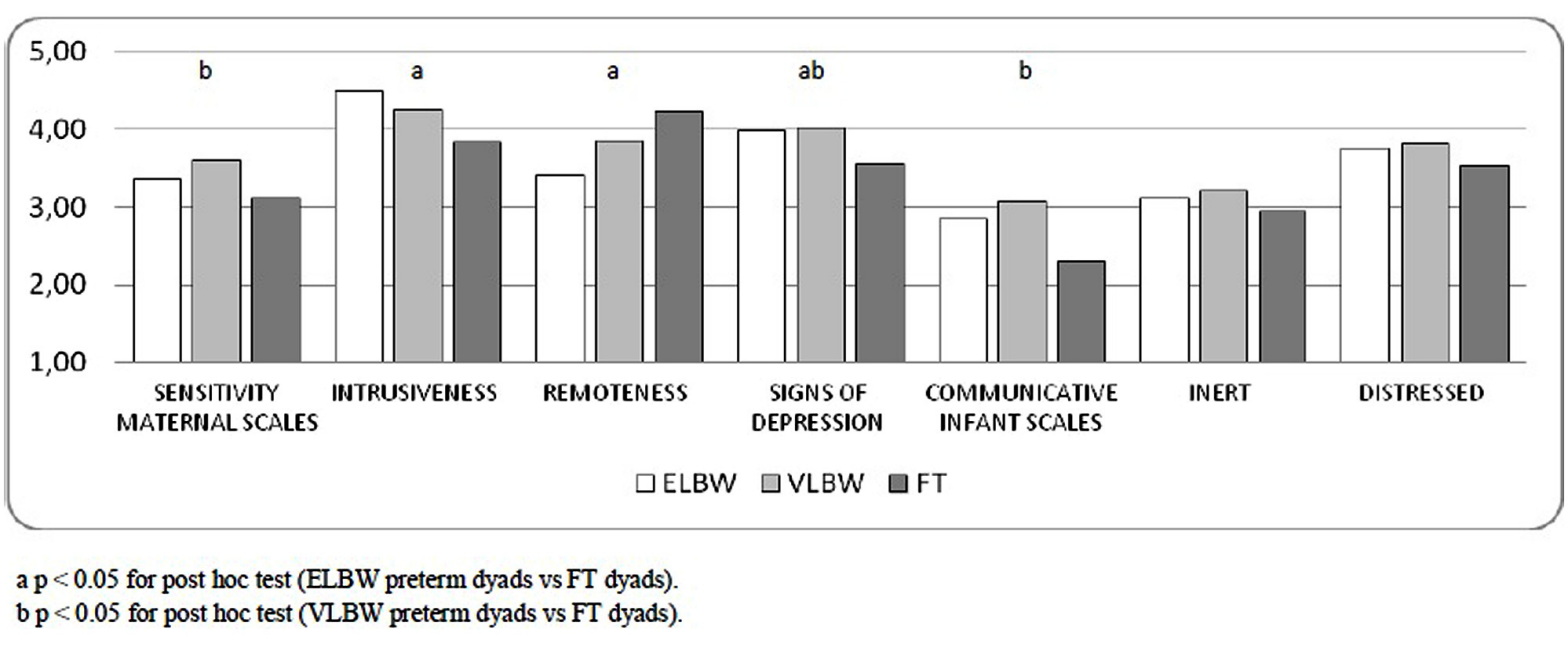
**Mother–infant interactive behaviors mean scores according to Birth Weight**.

In the case of Sensitivity dimension, VLBW mothers obtained higher scores than those of FT infants (Bonferroni *post hoc*, *p* = 0.031). When maternal Intrusiveness and Remoteness dimensions were considered, ELBW mothers showed more intrusive and less remote behaviors than the mothers of FT ones (Bonferroni *post hoc*, *p* = 0.006; *p* = 0.012). Finally, Bonferroni *post hoc* showed higher scores on Signs of Depression dimension in the mothers of ELBW and VLBW infants than those of FT infants (*p* = 0.028; *p* = 0.022, respectively; **Figure 1**).

When infant’s scales were considered, a significant effect of birth weight emerged on Communicative dimension [*F*(2,196) = 6.436; *p* = 0.002]: VLBW infants showed higher scores than those of FT infants (Bonferroni *post hoc*, *p* = 0.016). No significant effect on any other infant dimensions emerged (**Figure 1**).

#### Maternal Symptomatology: Depression and Anxiety

When we considered the categorical score of EPDS (depressed vs. non-depressed), 10.2% women (*n* = 20) of the total sample resulted in the “depressed group.”

As to mother–infant interactions, maternal depression showed a significant effect on the mean scores of Remoteness dimension [*F*(1,196) = 7.389; *p* = 0.007]: depressed mothers were more remote than non-depressed ones. Besides, depressed mothers showed a lower mean score on the Signs of depression dimension [*F*(1,149) = 5.373; *p* = 0.022; **Table 2**], meaning that their interactive behavior was significantly affected by their negative affective state in terms of low energy, sad facial expressions and tendency to self-absorption. No differences emerged between depressed and non-depressed groups on mothers’ Sensitivity and Intrusiveness dimensions and on any of the infants’ dimensions considered (**Table 2**).

Considering the categorical score of PSWQ (anxious vs. non-anxious), the “anxious group” was composed of 7.6% of the women (*n* = 15%) of the total sample.

With regard to mother–infant interaction, the analyses showed no differences between the two groups on the dimensions of Intrusiveness, Remoteness, and Sign of Depression (**Table 2**). Indeed, anxious mothers obtained lower mean scores than non-anxious ones as to Sensitive dimension [*F*(1,196) = 4.355; *p* = 0.038]: this means that they showed less ability to detect and understand the infant’s signals and to respond with adequate levels of acceptance, affection and warmth. No differences emerged between anxious and non-anxious groups on any dimensions of the infants’ interactive behavior (**Table 2**).

**FIGURE 2:**
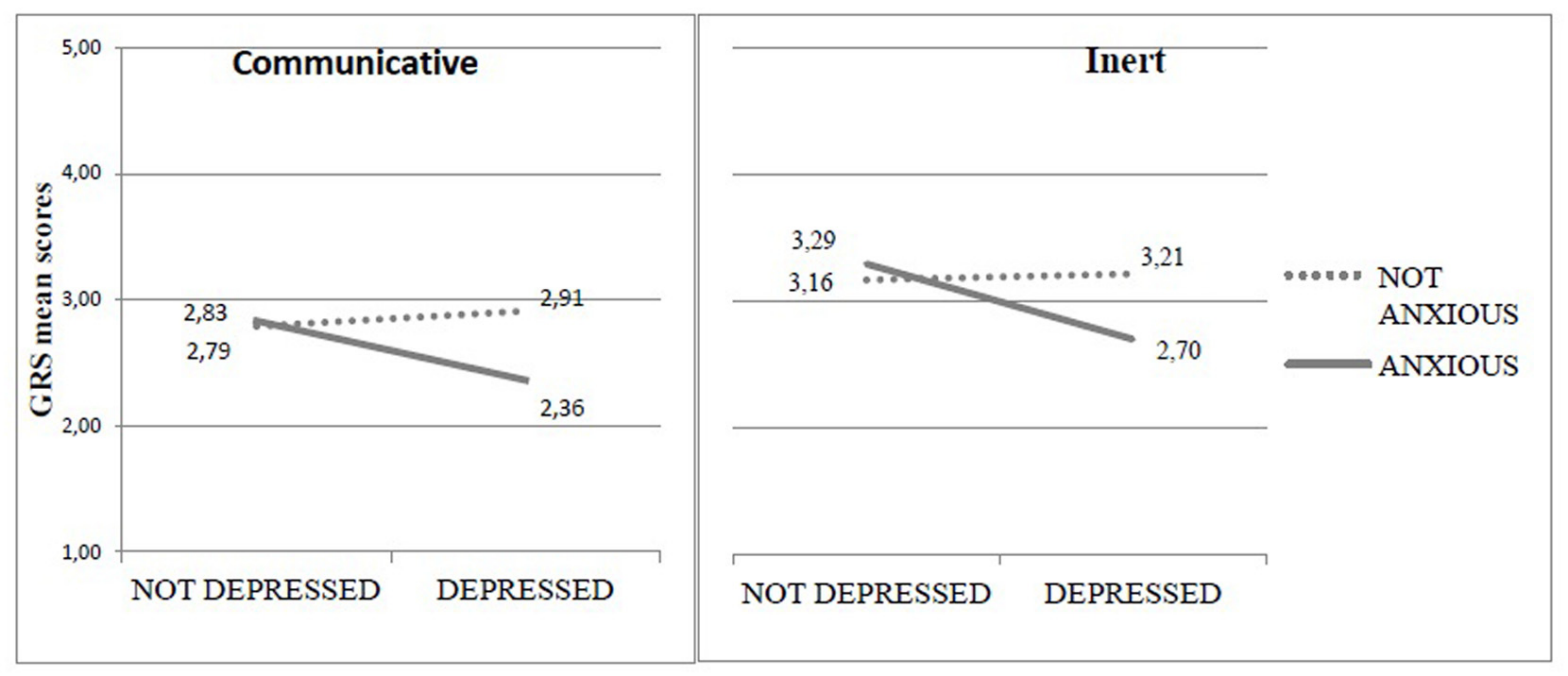
**Communicative and Inert mean scores in relation to depressive × anxious symptomatology**.

A significant interaction effect emerged between maternal depressive and anxiety symptomatology on the mean scores of Signs of Depression [*F*(1,196) = 3.726; *p* = 0.050], Communicative [*F*(1,196) = 6.041; *p* = 0.015], and Inert dimension [*F*(1,196) = 3.777; *p* = 0.050]. In all cases, the comorbidity of depression and anxiety was associated to lower levels of affective maternal behaviors and less attention, communication and engagement in the infant (**Figure 2**).

#### Birth Weight and Maternal Symptomatology

The three birth weight groups showed significant differences on EPDS mean scores [*F*(2,196) = 11.345; *p* = 0.001]: the mothers of ELBW infants showed higher scores compared to those of VLBW and FT ones (10.06, 6.62, 5.39, respectively; Bonferroni *post hoc* test *p* = 0.015 and *p* < 0.0005, respectively). The Pearson chi square test showed a significantly higher frequency of depressed women in the ELBW group compared to those of the FT group (25.0, 4.2%, respectively; χ^2^(2) = 13.888, *p* = 0.001); even if 15.6% of VLBW infants’ mothers were depressed, their prevalence did not significantly differ from the other two groups.

The interaction between birth weight and maternal depression showed no significant effect on any interactive dimensions mean scores.

When maternal anxiety was considered, the mean scores of PSWQ resulted significantly different among the three categories of birth weight [*F*(2,196) = 4.31; *p* = 0.026]: Bonferroni *post hoc* analysis showed that ELBW infants’ mothers got higher scores compared to FT infants’ ones (44.22, 37.85, respectively; *p* = 0.026). The Pearson chi square test showed a significantly higher prevalence of anxious women in the ELBW group compared to those in the VLBW and FT ones [21.9, 6.7, 4.2%, respectively; χ^2^(2) = 11.336, *p* = 0.003].

**FIGURE 3:**
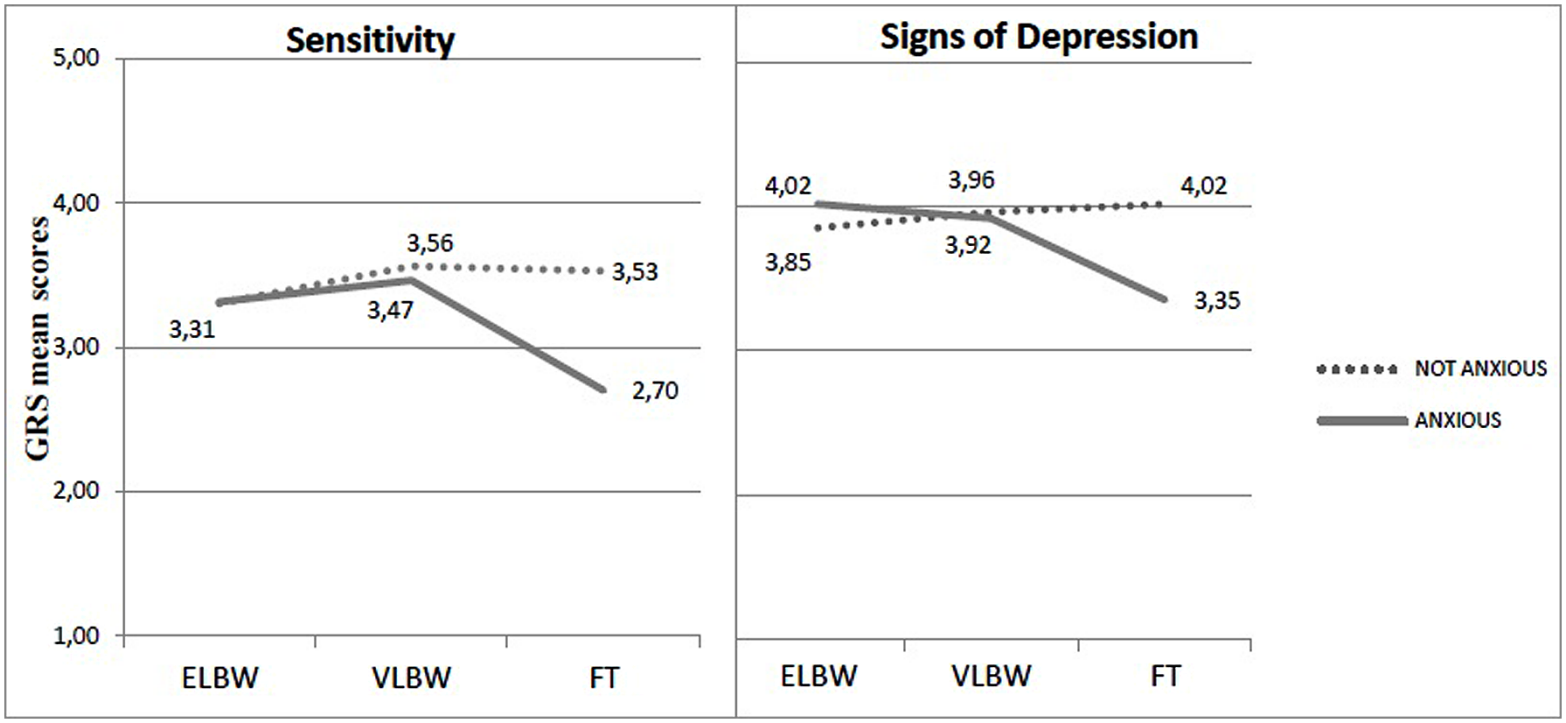
**Sensitivity and Signs of Depressions mean scores in relation to birth weight × anxious symptomatology**.

Differently from maternal depression, a significant interaction between birth weight and anxiety emerged on the means scores of Sensitivity, Signs of Depression and Communicative dimensions [*F*(2,196) = 5.513; *p* = 0.007; *F*(2,196) = 3.445; *p* = 0.034; *F*(2,196) = 5.768; *p* = 0.004, respectively; **Table 2**]. Simple effect analyses showed that, only in FT mothers, lower scores were strongly associated to the presence of anxious symptoms, while for ELBW and VLBW mothers the quality of interaction was similar, independently from the exhibition of anxiety symptoms (**Figure 3**).

## Discussion

Many studies have investigated the effect of maternal affective state on the quality of mother–infant interactions in the first 3 months of life, especially in the context of preterm birth. However, to our knowledge, there is a lack of studies exploring both the direct and the combined effect of maternal affective state (depressive and anxious symptoms) and the severity of preterm birth on early interactions. Therefore, we aimed at investigating how mother–infant interactions were influenced by maternal depression, anxiety and the severity of preterm birth.

A first interesting result is related to the effect of preterm birth on early interactions. Even if the development of mother–infant relationship in case of preterm birth has been deeply analyzed in literature, the effect of birth weight as a risk factor is still understudied. Our study has the strength of considering two groups of preterm infants characterized by a different level of severity of prematurity (very/extremely low birth weight) and a control group of full-term dyads.

In the present study, birth weight did not seem to compromise the quality of infant interactive patterns: preterm babies showed similar interactive scores compared to full-term infants; indeed, differently from what emerged from previous studies (Crawford, 1982; Singer et al., 2003; Korja et al., 2012; De Schuymer et al., 2012), they did not show high levels of passivity, fretful and disengaged behaviors. Moreover, VLBW infants appeared very communicative, with higher mean scores when compared to full-term ones.

When maternal interactive patterns are considered, many differences emerged as to birth weight. However, the results seem to indicate that birth weight has a different and specific effect on the single dimensions of maternal interactive behavior. Globally, all mothers showed good scores on Sensitivity dimensions, with higher mean scores in VLBW mothers compared to FT mothers. This result was somehow unexpected, because many authors previously described preterm babies’ mothers as non-sensitive (Zarling et al., 1988; Muller-Nix et al., 2004; Forcada-Guex et al., 2006, 2011). Nevertheless, other studies failed to find significant differences between maternal interactive behaviors when comparing preterm to FT mothers (Greenberg and Crnic, 1988; Schermann-Eizirik et al., 1997; Korja et al., 2008a; Montirosso et al., 2010), showing how prematurity was associated to high levels of caretaking (Crawford, 1982; Jean and Stack, 2012). The attention paid to different categories of prematurity may show specific interactive patterns, which did not emerge in previous studies where prematurity was considered as a global and homogenous category. The same explanation may be useful to understand the differences between VLBW and FT infants that emerged on infant communication dimensions. It is also important to consider the characteristics of the instrument chosen to evaluate interactive behaviors; GRS scales describe Sensitivity as the mother’s ability to detect and understand her infant’s signals and to appropriately respond, with adequate levels of acceptance, affection and warmth (Murray et al., 1996a). Therefore, our study may stress the differences related to the warmth and affection showed by VLBW mothers.

When the maternal intrusive and remote behaviors were considered, the results were consistent with previous studies (Crawford, 1982; Muller-Nix et al., 2004; Forcada-Guex et al., 2006; Korja et al., 2012) that described preterm mothers as very active and overstimulated. However, this pattern was detected only in the ELBW infants’ mothers. In the case of VLBW, the higher level of infant communication may possibly endorse maternal behaviors, reducing their intrusiveness. This result may add some information to the previous literature (Schmücker et al., 2005; Forcada-Guex et al., 2011) about the role of birth weight as a possible moderator between preterm birth and maternal intrusiveness, thus widening the areas for future investigation.

Another interesting result is related to the mean scores obtained by both groups of preterm infants’ mothers on the Signs of Depression dimension. This data may appear inconsistent with the prevalence of depression found by means of EPDS, which was significantly higher in ELBW mothers than in FT ones. As to the Sensitivity dimension, some considerations must be taken into account. The prevalence of depression detected with the EPDS concerns the subjective perception of maternal affective state as to sense of guilt, lack of pleasure, and dissatisfaction. To this end, the levels of depression found in ELBW infants’ mothers confirm and enrich the existing literature (Padovani et al., 2004; Miles et al., 2007; Vigod et al., 2010; Brandon et al., 2011; Gray et al., 2012), showing how the higher is the severity of prematurity the higher is the risk of maternal depression. On the contrary, the GRS Signs of Depression dimension focuses on detecting depressive symptoms emerged during the interaction with their baby in terms of low energy, self-absorption and poor engagement with the infant (Murray et al., 1996a). It should be noted that literature on prematurity shows preterm infants’ mothers to be engaged with their babies also in case of depression (Agostini et al., 2014): this pattern was observed in both groups of preterm dyads (ELBW and VLBW) and it might represent a specific interactive behavior of their mothers, while depression detected by EPDS was high only in the case of ELBW infants’ mothers. As a result, the difference found between the scores of the EPDS and the GRS Sign of Depression dimension might be explained as the different aspects of depression investigated by the two instruments. Moreover, it should be considered that while the EPDS is a self-report questionnaire, the GRS is a measure of the quality of interaction evaluated by a blinded rater: therefore he can observe also adequate interactive behaviors independently by the level of maternal sense of guilt, lack of pleasure, and dissatisfaction. This aspect has clinical implications: when interventions are planned to support parenting skills, the GRS may enrich maternal self-representation, showing positive aspects that could be missed by the mother due to her depressive mood/state.

Another objective of the study was to evaluate the effect of maternal symptomatology (depression and anxiety) on the quality of mother–infant interactions. The results seem to show that, when their interaction is considered, maternal depression was associated with Remoteness and Sign of Depression dimensions, while maternal anxiety significantly affected the level of Sensitivity.

The results seem to underline that both kind of symptoms impaired the relationship between mothers and their infants, but with a specific effect on maternal behaviors: depression has a negative influence on the maternal ability to stay close to her infant, on her level of energy and engagement during the interaction; conversely, anxiety has a major effect on the skills of perceiving and responding to infant cues. This result is very relevant and widens the literature on the topic, since previously depression and anxiety have been investigated separately.

Another peculiarity of the present study was the use of PSWQ to detect the symptoms of maternal anxiety. While in previous studies about perinatality, anxiety was often evaluated through generic instruments as STAI (Padovani et al., 2004, 2008; Correia and Linhares, 2007; Zelkowitz et al., 2007, 2009), we chose to focus on worries, a specific component of anxiety. It could be hypothesized that the tendency to worry or ruminate might alter the mother’s attentional focus, reducing her ability to adequately respond to her infant’s cues (Stein et al., 2012).

Interestingly, the study did not detect a direct effect of anxiety on any infant dimensions. However, when the interaction between maternal depression and anxiety was considered, we found a significant effect on infant Communicative and Inert dimensions: in both cases, the infant interactive behaviors were negatively influenced by the presence of maternal depression and anxiety. Besides, a significant interaction effect emerged on the Signs of Depression dimension, underlining how the level of maternal affective state, as measured by GRS scales, was affected by the presence of depression and worsened in case of comorbidity with anxiety – a cumulative effect.

Globally, the results seem to indicate a direct effect of maternal symptomatology (depression or anxiety) on maternal behaviors, whereas infant interactive patters seem to be more influenced by the co-occurrence of maternal depression and anxiety. Considering that the comorbidity of depression and anxiety is high in the first postpartum period (18–34%; Reck et al., 2008), it is very important that future studies pay particular attention to the effects of co-occurrence of both maternal depression and anxiety.

The study confirmed how the first postpartum months can be a very sensitive period with an elevate risk of onset of maternal depression and anxiety, especially in case of preterm birth. However, it is important to note that, when preterm birth was considered, a high prevalence of both depressive and anxious symptoms only in ELBW mothers were found, while in case of VLBW group the frequency was similar to those of full-term mothers.

As to depressive symptomatology, the review by Vigod et al. (2010) showed how a severe preterm birth may be associated, during the first postnatal year, with higher levels of depressive symptoms in LBW and VLBW infants’ mothers. This study deepened Vigod’s results adding the evaluation on ELBW infants’ mothers.

To our knowledge, there are no specific studies that investigate the prevalence of maternal anxiety according to preterm birth weight. In this research, the use of PSWQ showed a high tendency of worries or rumination only in ELBW infants’ mothers. The extreme severity of prematurity may possibly represent a very frightful event for mothers with concerns and worries which last for many months after the discharge of the baby (Singer et al., 1999; Wijnroks, 1999; Kersting et al., 2004; Feeley et al., 2007).

Considering both depression and anxiety, the results give a contribution to literature and it emphasizes the need for future research to distinguish among different preterm conditions. This element could deepen existing literature and identify those parents who, after a preterm birth, are forced to face stress with less adaptive coping skills.

However, it should be noted that, in full-term mothers, the rate of maternal depressive and anxiety symptoms was slightly lower compared to the prevalence reported in other studies (depression: 4.2 vs. 10%; Murray et al., 1996a; Gavin et al., 2005; anxiety: 4.2 vs. 27.9%, Ross et al., 2003; Britton, 2005). This result could be partially influenced by the method of recruitment and explained by the sample characteristics; therefore, it is suggested to confirm these data on wider samples in the future.

According to the objectives of this study, an interactive significant effect emerged between the birth weight and maternal symptomatology only in case of maternal anxiety. As previously reported, the use of PSWQ could be particularly adequate to detect how the tendency to worry may impair the maternal ability to capture and reply to their infant’s needs (Stein et al., 2012). Interestingly, this effect is particularly evident in the mothers of the control group. In absence of birth risk factor, this group of mothers tends to interact with the infant in a sensitive way. The presence of anxious symptomatology can worsen the maternal ability to interact with the infants, while maternal anxiety may represent a very common condition when premature birth occurs. The influences of anxiety on mother–infant interactions could be then screened by the preterm birth, while anxiety may function as an adaptive response in this traumatic condition to help mothers to maintain the focus on the infant and their relationship.

Differently, depression have the same negative effects on preterm and FT dyads, confirming existing literature (Murray et al., 1996a; Korja et al., 2008b; Poehlmann et al., 2011).

Some considerations must be done regarding the NICU where preterm infants were recruited. According to NIDCAP guidelines (Als and Gibes, 1986; Ohlsson and Jacobs, 2013), all the staff is careful to protect and enhance the infant’s and parents’ quality of life. Since the first moments of life, the relationship between preterm infant and their parents is guaranteed by 24-h free access to the unit. In fact, after preterm birth mothers could feel inadequate to care to such a fragile infant, a number of specialized professional figures (e.g., physiotherapists, clinical psychologists) are present to help them feel useful and involved in their infant’s care. To this end, many methods are used, such as kangaroo care, infant massage, both useful to teach to touch the infant in a comfortable way, and reading a little fairy-tale, which allow the baby to listen to maternal voice as it used to do during pregnancy (Biasini et al., 2015a). Literature states that these interventions are very important to reduce the level of psychological distress in the NICU and after discharge (Melnyk et al., 2006; Trombini et al., 2008), with positive effects on the quality of the dyadic interaction. These elements could explain the high levels of maternal sensitivity and infant communicative interactive patterns found in this study. These protective factors could be particularly relevant for the VLBW group, which being less at-risk may better benefit from the interventions. Globally, when a NICU functions as a supportive environment, it can promote positive mother–infant interactions during hospitalization, inducing mechanistic changes in the brain structure and function, maximizing positive neurodevelopmental outcomes and reducing neurologic deficits (Weber et al., 2012).

Some limits of the study might be acknowledged. Firstly, all results need replication, based on studies with wider samples. Secondly, in the present study the role of the infant’s father was not considered. Because the father plays a relevant role in moderating the effects of maternal depression on dyadic interactions and can represent a strong source of support for his partner (Robertson et al., 2004; Fletcher, 2009), it is advisable that future studies should deepen this. Moreover, further investigation could better understand the possible influences of maternal characteristics, i.e., parity or level of education. Finally, it will be clinically relevant to understand how these mother–infant interactions will evolve in a longitudinal way.

## Conclusion

Globally, the results suggested that mothers of both preterm groups’ appear involved and close to their babies. Nevertheless, the ELBW preterm group was characterized by a higher risk of maternal symptomatology and by a more “intrusive and controlling” mothering. These results, therefore, seem to suggest that, within preterm populations, the ELBW dyads could represent a sample with peculiar characteristics, specific needs and difficulties. So, during ELBW babies’ hospitalization and at the moment of their discharge, hospital staff should pay special attention to both the infant’s development and the parental affective state, in order to prevent the onset of depression or anxiety and to give a prompt intervention. Specifically, *ad hoc* interventions should be promoted to assess the risk of depressive or anxiety symptoms with adequate tools to give special support and treatment for symptomatology and to enhance parental functioning.

The longitudinal evaluation of mother–infant interaction in these dyads will help give a more comprehensive description of the long-term effects of depressive and anxious symptoms on interactive patterns, and if birth weight could act as a moderator in the relationship between maternal and infant interactive patterns.

## Conflict of Interest Statement

The authors declare that the research was conducted in the absence of any commercial or financial relationships that could be construed as a potential conflict of interest.
